# Prevention of pruritus with ethyl-chloride in skin prick test: a double-blind placebo-controlled prospective study

**DOI:** 10.1186/s13223-015-0091-z

**Published:** 2015-09-04

**Authors:** Amir Gal-Oz, Shmuel Kivity, Yacov Shacham, Elisheva Fiszer, Ori Rogowsky, Gil Chernin

**Affiliations:** Nephrology Department and Medicine, Tel-Aviv Sourasky Medical Center, Sackler School of Medicine, Tel-Aviv University, 6, Weizmann Street, 64239 Tel Aviv, Israel; Allergy Department, Tel-Aviv Sourasky Medical Center, Sackler School of Medicine, Tel Aviv University, Tel Aviv, Israel; Internal Medicine Department G Tel-Aviv, Tel-Aviv Sourasky Medical Center, Sackler School of Medicine, Tel-Aviv University, Tel Aviv, Israel

**Keywords:** Ethyl-chloride spray, Histamine skin-prick test

## Abstract

**Background:**

Ethyl-chloride (EC) spray was recently shown to be an effective antipruritic agent, when given 15 min after histamine skin-prick test (SPT), without changing the wheal and flare reaction. We aimed to investigate the antipruritic effect of EC on SPT, when given prior to SPT.

**Methods:**

A double-blind placebo-controlled prospective study. Overall, 44 volunteers underwent histamine SPT on both arms to trigger local pruritus. Prior to test, they were randomly treated with EC spray on one arm and saline spray (placebo) on the other. Subjects as well as researchers were blinded to the type of applied sprays. The wheal and flare reaction was measured after the SPT and subjects reported the intensity of pruritus following EC/placebo using a validated pruritus questionnaire (indexes 1–3) and a visual analog scale (VAS).

**Results:**

Significant improvement in pruritus was reported following treatment with EC compared with placebo for all four studied parameters. Index 1 in EC 3.7 ± 2.3 versus 5 ± 3.5 (p = 0.007) in placebo, index 2 in EC 2.6 ± 2.1 versus 3.8 ± 2.8 (p = 0.002) in placebo, index 3 of EC 6.3 ± 3.8 versus 8.8 ± 5.8 (p = 0.03) and VAS in EC 3.7 ± 1.9 versus 4.4 ± 2.3 (p = 0.003). There were no significant differences between EC and placebo in terms of the wheal and flare indurations area.

**Conclusions:**

Ethyl-chloride has an effective antipruritic agent, when given before histamine SPT. Its use did not change the wheal and flare reaction, making it ideal for prevention of pruritus, secondary to allergy skin test, without masking the results.

## Background

Skin prick tests (SPT) for the diagnosis of IgE mediated allergy are relatively safe procedures. Nevertheless, it may cause severe sensation of either pain or pruritus in both children and adults [1, 2]. Therefore, measures to prevent or reduce these symptoms may be warranted. Topical agents such as eutectic mixture of local anesthetics (EMLA; AstraZeneca LP, Wilmington, DE), have been shown to improve symptoms with skin testing, yet these agents may mask the wheal and flare response areas, thus limiting its usefulness [2–5].

Ethyl-chloride (EC) is an anesthetic agent used in a vapo-coolant spray for topical anesthesia in minor surgical procedures, minor sports injuries, pre-injection anesthesia and myofascial pain [1, 6]. Scarce data exist on the potential effects of topical EC spray on pain and pruritus in skin allergy testing and whether its use may mask the wheal and flare indurations areas. Applying topical EC prior to SPT did not significantly reduce the pain sensation although a trend toward pain alleviation was observed [1]. We have shown that EC spray was effective as an antipruritic agent when given after histamine SPT [7]. Topical EC was not associated with changes in the wheal and flare reaction [1, 7]. Only few case reports of allergic contact dermatitis from EC and a positive patch testing with EC were previously published [8–10]. Considering its widespread use as a local anesthetic, it is probably an uncommon adverse event of EC spray.

In the current double-blind placebo-controlled prospective study, we aimed to investigate the antipruritic effects of EC-spray, when given prior to the histamine SPT and whether its use may change the wheal and flare reactions.

## Methods

### Study design

A double-blind placebo-controlled prospective study was conducted between February 2013 and October 2013. The study protocol was approved by the local ethics committee, and the participants signed an informed consent with permission to publish.

### Included and excluded subjects

Included were adult volunteers (age >18 years), willing to participate in study.

Exclusion criteria: (1) subjects with active skin disease or factitious urticaria. (2) Patients treated with analgesics, other medications that may affect pain such as (e.g., Gabapentin), antihistamines or systemic steroids within 7 days prior to the experiment. (3) Patients with neuropathic disorders or dermatographism. (4) Subjects with a history of oncological disease. (5) Immunocompromised patients, not including controlled diabetes mellitus (DM). (6) Breastfeeding women. (7) Subjects with active skin disease, and (8) subjects with current or recent (<7 days) febrile disease.

### Study protocol

All subjects underwent the histamine SPT on both arms in order to trigger local pruritus, by the same well trained physician. Histamine was introduced in increasing concentrations on three spots along both forearms: 2.5, 5.0 and 10.0 mg/ml. Normal saline was used as negative control on fourth spot. Prior to SPT, The affected areas were treated with EC (Gebauer, Cleveland, OH, USA) spray on one arm and saline spray (placebo) on the other.

Ethyl-chloride was applied per package recommendation (i.e. for 3–7 s at a distance of about 15 cm, just until the skin began to frost) 5 s before initiation of histamine SPT. Placebo spray was applied for the same time and at the same distance on the other arm using a sprayer that mimicked the EC-spray. Subjects as well as researchers were blinded to the sprays used. The wheal and flare reactions were measured by the same researcher (A.G.O.) 15 min following SPT. The borders of the indurations area were marked and copied to a clear tape. Then the wheal area was measured (in mm^2^). Subjects rated pruritus by using a visual analog scale (VAS) and a pruritus questionnaire (see below), 15 min following the histamine SPT.

### Pruritus questionnaire

Despite the clinical importance, there is no validated questionnaire for the evaluation of pruritus. The questionnaire we used was based on a pruritus questionnaire previously validated in uremic patients by Yosipovitch et al. [7, 11]. We used only part of the referenced questionnaire which is relevant for the evaluation of the sense of itching—the verbal descriptor scale of itch sensation and ‘the severity of pruritus’. It included three indexes. In index 1, subjects were asked to rank the severity of six pruritus sensations (‘tickling’, ‘stinging’, ‘crawling like ants’, ‘stabbing’, ‘pinching’ and ‘burning’) on a scale from 0 (=none) to 3 (=severe), and these ranks were summed. Hence, the index 1 final score ranged between 0 and 18. In index 2, subjects were asked to rank the severity of four pruritus dimensions (‘bothersome’, ‘annoying’, ‘unbearable’ and ‘worrisome’) on a scale from 0 (=none) to 3 (=severe), and these ranks were summed. Hence, the index 2 final score ranged between 0 and 12. Index 3 included the sum of both prior indexes. Hence, it ranged between 0 and 30.

### Visual analog scale

The VAS is usually used to evaluate pain intensity. In this study it was used to evaluate pruritus intensity. It consisted of a 10-cm line anchored at one end by the label ‘no itch’ and at the opposite end by the label ‘very strong itch, as bad as could possibly be’. Pruritus was ranked from 1 (no pruritus) to 10 (maximal pruritus).

### Statistical analysis

Continuous variables were expressed as means and standard deviation. The one-way Kolmogorov–Smirnov test was used to assess the distributions of parametric variables. The Paired t test was used to compare mean VAS scores and mean pruritus indexes between the arms treated with EC and arms treated with placebo. A p value of less than 0.05 was considered statistically significant (two sided). Version 21.0 of the SPSS statistical package (SSPS Inc., Chicago, IL, USA) was used to perform all the statistical evaluations.

## Results

Forty-four healthy volunteers (males = 19; females = 25) underwent histamine-SPT.

Age-range was 19–57 years. The basic clinical characteristics are summarized in Table 1. Significant medical history was noted in only four patients. One volunteer had a history of skin disease (psoriasis) that was not active for 3 years. One volunteer had well controlled DM type 2 without clinical signs of diabetic neuropathy. Only one volunteer had more than one significant chronic disease with well controlled hypertension and hypothyroidism.

**Table 1:** Clinical characteristics of 44 volunteers in study

Sex (m/f)	19/25
Age	
Range (years)	19–57
Mean ± SD (years)	32 ± 7.4
Ethnicity	
Caucasians	44 (100 %)
Other	0 (0 %)
Medical History (number of individuals)	Hypertension (1) Hypothyroidism (2) Non-active psoriasis (1) Diabetes mellitus type 2 (1)
Medications	Amlodipine (1) Levothyroxine (2) Metformin (1)

All four studied parameters (index 1, index 2, index 3 and VAS) distributed normally among the whole cohort and among both genders. Age and gender were not associated with these parameters in a multivariate analysis.

**Fig. 1:**
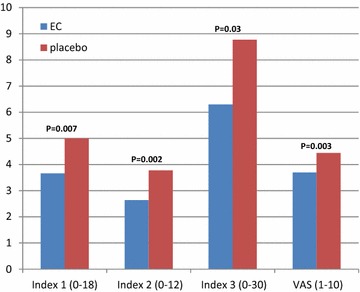
Effect on pruritus caused by histamine skin prick test with pretreatment of ethyl-chloride (EC) or placebo. Fifteen minutes after histamine skin prick test, there were statistically significant differences between arms in favor of EC over placebo in all four studied parameters of pruritus

Fifteen minutes after histamine-SPT, there were statistically significant differences between arms in favor of EC over placebo in all four studied parameters of pruritus (Fig. 1). Index1 in EC 3.7 ± 2.3 versus 5 ± 3.5 (p = 0.007) in placebo, index 2 in EC 2.6 ± 2.1 versus 3.8 ± 2.8 (p = 0.002) in placebo, index 3 of EC 6.3 ± 3.8 versus 8.8 ± 5.8 (p = 0.03) and VAS in EC 3.7 ± 1.9 versus 4.4 ± 2.3 (p = 0.003).

**Fig. 2:**
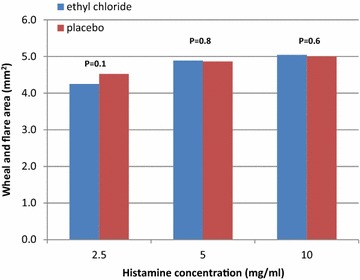
Effect of preventive treatment with ethyl-chloride (EC) or placebo on the wheal and flare indurations area following histamine skin prick test. For all the three different histamine concentrations, there were no significant differences between arms treated with EC or placebo in the indurations areas of the wheal and flare reactions

For all the three different histamine concentrations, there were no significant differences between arms treated with EC or placebo in the indurations areas of the wheal and flare reactions, 15 min following the histamine-SPT (Fig. 2). In concentration 2.5 mg/ml in EC 4.3 + 0.9 versus 4.5 ± 0.8 mm^2^ in placebo (p = 0.1), in concentration 5.0 mg/ml in EC 4.9 ± 0.7 versus 4.9 ± 0.6 mm^2^ in placebo (p = 0.8) and in concentration 10 mg/ml in EC 5.04 ± 0.8 versus 5.0 ± 0.8 mm^2^ in placebo (p = 0.6).

No significant side effects were reported during the study.

## Discussion

We found that applying topical EC spray, prior to histamine-SPT, reduced significantly the symptoms of pruritus in comparison with placebo (topical saline). In addition, EC spray was free of adverse effects and did not reduce the wheal and flare reactions. Taken together, our results suggest that EC spray may be ideal for prevention of pruritus secondary to allergy skin testing, without masking the SPT results. Although tested here on adult volunteers, EC prevention may specifically benefit children referred to SPT and indeed EC is safe and approved for the pediatric population [12, 13]. To the best of our knowledge, this is the first study that examined EC-spray as an antipruritic preventive measure before SPT. We have previously shown, in a double-blind placebo-controlled prospective study, that EC spray is an effective antipruritic agent when given 15 min after histamine SPT [7]. EC spray also did not change the wheal and flare reactions [7]. Waibel et al. conducted a randomized, double masked, placebo-controlled study on 18 individuals to evaluate the effect of EC spray on pain, caused by SPT. SPT were performed within 5–10 s of EC spray application. The main findings were that EC spray did not reduce the histamine and aeroallergen wheal and flare indurations areas during SPT [1]. Pain was reduced in some patients yet without statistical significance in comparison with the placebo group [1]. Pain and pruritus sensations may have some similar biological components and both may be triggered by histamine, yet they differ in some biological and physiological aspects [14, 15]. It may well be that the different symptoms of pain and pruritus respond differently to EC-spray [1, 7]. It is also possible, that the difference in sample size between the study of Waibel et al. and the current study, contributed to the significant reduction in pruritus described here.

Histamine is released from mast cells when they are activated under various inflammatory conditions [14, 15]. Possible explanation for the pruritus induced by histamine was proposed by Han et al. who demonstrated that activation of histamine H1 receptor on C fibers by histamine may induce itching by activation of phospholipase CB3 that links G protein-coupled receptors to an intracellular signaling network [16]. There are few possible mechanisms by which EC may alleviate pruritus: (1) direct local analgesic effect of EC on dedicated C fibers. These fibers can sense temperature changes and EC spray may inhibit pruritus by topical temporal freezing [17, 18]. (2) As suggested by the ‘gate theory’ of pain, central processing of the pruritus mechanism, both itch and pain, can be reduced by soft rubbing, which activates fast-conducting, low-threshold fibers [19]. It is therefore possible that pruritus reduction is based on a spinal antagonism between thermal and itch-processing neurons. The cold sensation caused by EC spray and stimulating the nerve does not allow the stimulus of pruritus sensation to be conducted. (3) Alleviating pruritus, even temporarily, may affect longer improvement of pruritus. In contrast to pain, removing the cause of pruritus does not necessarily stop the itch and might even aggravate it (‘itch-scratch-itch cycle’ theory) [20]. In the current study, EC was applied before the SPT and therefore it does not support this theory. (4) Local vasoconstriction by EC might contrast the vasodilatation caused by histamine, and in addition cooling inhibits activation of nociceptors. However, we believe that this possible antipruritic mechanism is the least likely explanation since there was no influence of EC on the wheal and flare induration area size.

Our study has limitations. It involves prevention of pruritus in histamine-SPT and not with other potential agents that cause pruritus. Therefore, one cannot draw conclusions about a general antipruritic effect of EC with other pruritogenic agents. Although participants in this study were blinded to the spray used, it is still possible that the transient cooling sensation of EC was recognized or at least suspected by the subjects. It may have influenced the description of itching in the pruritus questionnaire and VAS. It is also important to notice that only adults were tested here and a study on the antipruritic effect of EC in children is needed.

## Conclusion

Prevention and improvement of pruritus, caused by histamine-SPT, is feasible with topical EC spray without masking the SPT results. It may be considered as an option to reduce pruritus discomfort of SPT.

## Abbreviations

EC: ethyl-chloride
DM: diabetes mellitus
SPT: skin-prick test
VAS: visual analog scale

## References

[1] Waibel KH, Katial RK (2005). Effect of topical vapocoolant spray on skin test wheal, flare, and pain responses. Ann Allergy Asthma Immunol.

[2] Sicherer SH, Eggleston PA (1997). EMLA cream for pain reduction in diagnostic allergy skin testing: effects on wheal and flare responses. Ann Allergy Asthma Immunol.

[3] Shuttleworth D, Hill S, Marks R, Connelly DM (1988). Relief of experimentally induced pruritus with a novel eutectic mixture of local anaesthetic agents. Br J Dermatol.

[4] Simons FE, Gillespie CA, Simons KJ (1992). Local anaesthetic creams and intradermal skin tests. Lancet.

[5] Pipkorn U, Andersson M (1987). Topical dermal anaesthesia inhibits the flare but not the weal response to allergen and histamine in the skin-prick test. Clin Allergy.

[6] Reis EC, Roth EK, Syphan JL, Tarbell SE, Holubkov R (2003). Effective pain reduction for multiple immunization injections in young infants. Arch Pediatr Adolesc Med.

[7] Gal-Oz A, Rogowski O, Swartzon M, Kivity S (2010). Ethyl chloride as an antipruritic agent: a double-blind placebo-controlled prospective study. Dermatology.

[8] Carazo JL, Morera BS, Colom LP, Gálvez Lozano JM (2009). Allergic contact dermatitis from ethyl chloride and benzocaine. Dermatitis.

[9] Kriechbaumer N, Hemmer W, Focke M, Götz M, Jarisch R (1998). Sensitization to ethyl chloride in a handball player. Contact Dermat.

[10] Aberer W, Zonzits E (1989). Allergy to ethyl chloride does occur, and might frequently be misdiagnosed. Contact Dermat.

[11] Yosipovitch G, Zucker I, Boner G, Gafter U, Shapira Y, David M (2001). A questionnaire for the assessment of pruritus: validation in uremic patients. Acta Derm Venereol.

[12] Soueid A, Richard B (2007). Ethyl chloride as a cryoanalgesic in pediatrics for venipuncture. Pediatr Emerg Care.

[13] Davies EH, Molloy A (2006). Comparison of ethyl chloride spray with topical anaesthetic in children experiencing venepuncture. Paediatr Nurs.

[14] Ikoma A, Cevikbas F, Kempkes C, Steinhoff M (2011). Anatomy and neurophysiology of pruritus. Semin Cutan Med Surg.

[15] Ikoma A (2013). Updated neurophysiology of itch. Biol Pharm Bull.

[16] Han SK, Mancino V, Simon MI (2006). Phospholipase Cbeta 3 mediates the scratching response activated by the histamine H1 receptor on C-fiber nociceptive neurons. Neuron.

[17] Mizumura K, Koda H (1999). Potentiation and suppression of the histamine response by raising and lowering the temperature in canine visceral polymodal receptors in vitro. Neurosci Lett.

[18] Schmelz M, Schmidt R, Bickel A, Handwerker HO, Torebjörk HE (1997). Specific C-receptors for itch in human skin. J Neurosci.

[19] Bromm B, Scharein E, Vahle-Hinz C (2000). Cortex areas involved in the processing of normal and altered pain. Prog Brain Res.

[20] Yosipovitch G, Papoiu AD (2008). What causes itch in atopic dermatitis?. Curr Allergy Asthma Rep.

